# Genomic regions underlying susceptibility to bovine tuberculosis in Holstein-Friesian cattle

**DOI:** 10.1186/s12863-017-0493-7

**Published:** 2017-03-23

**Authors:** Kethusegile Raphaka, Oswald Matika, Enrique Sánchez-Molano, Raphael Mrode, Mike Peter Coffey, Valentina Riggio, Elizabeth Janet Glass, John Arthur Woolliams, Stephen Christopher Bishop, Georgios Banos

**Affiliations:** 1 The Roslin Institute and Royal (Dick) School of Veterinary Studies, University of Edinburgh, Easter Bush, Midlothian, EH25 9RG Scotland, UK; 2 Scotland’s Rural College, The Roslin Institute Building, Easter Bush, Midlothian, EH25 9RG Edinburgh, UK

**Keywords:** Bovine tuberculosis, Susceptibility, Genome-wide association, Regional heritability mapping, Chromosome association

## Abstract

**Background:**

The significant social and economic loss as a result of bovine tuberculosis (bTB) presents a continuous challenge to cattle industries in the UK and worldwide. However, host genetic variation in cattle susceptibility to bTB provides an opportunity to select for resistant animals and further understand the genetic mechanisms underlying disease dynamics.

**Methods:**

The present study identified genomic regions associated with susceptibility to bTB using genome-wide association (GWA), regional heritability mapping (RHM) and chromosome association approaches. Phenotypes comprised de-regressed estimated breeding values of 804 Holstein-Friesian sires and pertained to three bTB indicator traits: i) positive reactors to the skin test with positive post-mortem examination results (phenotype 1); ii) positive reactors to the skin test regardless of post-mortem examination results (phenotype 2) and iii) as in (ii) plus non-reactors and inconclusive reactors to the skin tests with positive post-mortem examination results (phenotype 3). Genotypes based on the 50 K SNP DNA array were available and a total of 34,874 SNPs remained per animal after quality control.

**Results:**

The estimated polygenic heritability for susceptibility to bTB was 0.26, 0.37 and 0.34 for phenotypes 1, 2 and 3, respectively. GWA analysis identified a putative SNP on *Bos taurus* autosomes (BTA) 2 associated with phenotype 1, and another on BTA 23 associated with phenotype 2. Genomic regions encompassing these SNPs were found to harbour potentially relevant annotated genes. RHM confirmed the effect of these genomic regions and identified new regions on BTA 18 for phenotype 1 and BTA 3 for phenotypes 2 and 3. Heritabilities of the genomic regions ranged between 0.05 and 0.08 across the three phenotypes. Chromosome association analysis indicated a major role of BTA 23 on susceptibility to bTB.

**Conclusion:**

Genomic regions and candidate genes identified in the present study provide an opportunity to further understand pathways critical to cattle susceptibility to bTB and enhance genetic improvement programmes aiming at controlling and eradicating the disease.

**Electronic supplementary material:**

The online version of this article (doi:10.1186/s12863-017-0493-7) contains supplementary material, which is available to authorized users.

## Background

Bovine tuberculosis (bTB) is a chronic disease caused by *Mycobacterium bovis (M. bovis)* and usually manifests with tuberculous lesions predominantly in the respiratory tract, although lesions could also be found elsewhere [1]. Despite the implementation of nationwide compulsory bTB eradication schemes that were introduced in the United Kingdom in 1950 [2], the incidence of bTB has been marked by a general upward trend since the 1990s [3] resulting in large financial losses for the bovine industry. In Great Britain, the greatest impact of animal and financial losses are experienced in South-Western England and Wales [4]. During 2010/2011, an estimated £152 million was spent on management and control of the disease in these areas [5]. Scotland was certified officially free of bTB (OTF) in 2009 [6].

In Great Britain, bTB control and eradication programme involves routine testing and compulsory slaughter of infected animals and cattle movement restrictions in the affected herds. Routine testing is based on the administration of the single intradermal comparative cervical tuberculin (SICCT) or ‘skin’ test to each animal, which entails simultaneous injection of both *M. bovis* and *M. avium* tuberculins side-by-side into the skin of the neck, followed by examination for evidence of localised inflammation after 72 h. Interpretation of the test follows a standard procedure applied internationally [7]. When reaction to *M. bovis* tuberculin injection is estimated to be less than or equal to that to *M. avium* tuberculin injection then the skin test is deemed negative. A positive skin test result, also known as a ‘reactor’, is asserted when the reaction to *M. bovis* tuberculin exceeds that to *M. avium* tuberculin by more than 4 mm. In all other cases, the test is considered inconclusive and retesting is done at 60-day intervals to resolve their status. A breakdown (bTB incident) is declared once at least one reactor is discovered in a herd, prompting animal movement restrictions, suspension of the OTF status of the herd and testing of all animals in the herd at 60-day interval. Animals with a positive or two consecutive inconclusive skin tests are slaughtered and examined at the abbatoir for visible lesions of bTB in their organs. Samples of tissue from a representative number of infected animals from each breakdown are sent to the laboratory where *M. bovis* culture is performed. A positive post-mortem examination result, i.e. presence of lesions and/or positive *M. bovis* culture (confirmed case) elicits a change of the herd’s OTF status from ‘suspended’ to ‘withdrawn’. The breakdown remains ‘open’ and skin testing continues in the herd until two consecutive negative herd tests are obtained.

Given the difficulties in eradicating bTB, breeding for resistance has been considered as an additional complementary control measure [8]. Most of earlier research on bTB was mainly focused on environmental risk factors for bTB infection [9–11], whilst limited attention was given towards identifying possible genetic factors in the bovine host. However, it was not until recently that genetic studies established the presence of between animal variation in dairy and beef cattle susceptibility to the disease with heritability estimates ranging between 0.09 and 0.23 [12–16]. Furthermore, some genome-wide association (GWA) and regional heritability mapping (RHM) analyses aiming at identifying Quantitative Trait Loci (QTL) underlying cattle susceptibility to bTB have been undertaken. GWA analysis by Finlay et al. [17] and Richardson et al. [18] identified genomic regions associated with bTB susceptibility on *Bos taurus* autosomes (BTA) 22 and 23, respectively, in Irish Holstein-Friesian dairy cattle. Bermingham et al. [19] found regions on BTA 13 in Northern Irish Holstein-Friesian dairy cattle using both GWA and RHM approaches. Tsairidou et al. [20] applied RHM to perform a meta-analysis using the datasets from previous studies in the Republic of Ireland [17] and Northern Ireland [19], and identified a new region on BTA 6. Furthermore, Kassahun et al. [21] also identified a SNP on BTA 6 associated with bTB in a mixed breed cattle population in Ethiopia; however, this region was distinct from that of Tsairidou et al. [20]. In general, genomic studies performed to date have not revealed any major common QTL; therefore further studies with independent populations are required.

Our objective was to conduct a first study of the genomic architecture of susceptibility to bTB in the British Holstein-Friesian cattle population. We used GWA, RHM and chromosome association approaches to analyse alternative definitions of bTB susceptibility that have not been genomically addressed before.

## Methods

### Phenotypes

Data for the present study were sire genetic evaluations that had been previously generated from the official genetic and genomic evaluation system for bTB resistance [15, 22]. These genetic evaluations had been based on skin test and post-mortem examination records of Holstein-Friesian cows obtained from breakdowns (herds with bTB incidents) that occurred between the years 2000 and 2014. Susceptibility to bTB was based on the health status of each animal in a breakdown, i.e. either infected (case) or healthy (control). Three alternative definitions of “infected” from Banos et al. [15] were considered:

i) Phenotype 1: positive reactors to the skin test with positive post-mortem examination results consisting of visible lesions of bTB and/or positive *M. bovis* culture. This phenotype represented the conservative definition of infected, which requires infection to be confirmed by post-mortem examination.
ii) Phenotype 2: positive reactors to the skin test regardless of post-mortem examination results, based on the very high specificity of the skin test (ca. 99%) and the trivial number of false positives expected [7]. Phenotype 2 included all phenotype 1 animals and those without positive post-mortem examination results.
iii) Phenotype 3: as in (ii) plus non-reactors and inconclusive reactors to the skin test who had been slaughtered and had positive post-mortem examination results, in order to include possible false negative skin tests in this definition [8]. The majority (97.3%) of this phenotype included phenotype 2 animals plus a few inconclusive (2.6%) and non-reactors (0.1%) to the skin test.

In all cases, healthy animals were defined as live non-reactors to the skin test or slaughtered non-reactors with negative post-mortem examination results. Animals defined as healthy were all from the same breakdowns as the infected ones.

Following the above trait definitions, a linear mixed model was used to calculate sire EBVs based on the phenotypes of their daughters. Each sire received three EBVs, one for each of the above trait definitions. More information about the genetic model used to derive these sire EBVs may be found in Banos et al. [15]. In the current study, sire EBVs were deregressed and used as phenotypes. The deregression was necessary because actual EBVs have been found to be unsuitable phenotypes for GWAS as they are usually regressed depending on pedigree structure and number of daughters per sire, and also include familial information all of which have the potential to reduce power, increase the rate of false positive results and misestimate QTL effect size [23]. The de-regression process accounted for sire EBV reliability and parental average effects, and followed the procedure described by Garrick et al. [24]. Consistent with the common genetic evaluation practice, de-regression was applied to sire EBVs with a minimum reliability of 0.30.

### Genotypes

Whole-genome genotypes based on the 50 K SNP Illumina BeadChip were available for 804 Holstein-Friesian sires with de-regressed EBVs for susceptibility to bTB. Genotype data were subjected to quality control using the software PLINK [25]. Quality control removed SNPs with minor allele frequency below 0.05 and call rates below 0.90, and significantly deviated from Hardy-Weinberg equilibrium (P < 1 × 10^−6^). Quality control also removed animals with individual call rates below 0.90. A total of 34,874 autosomal SNPs and 803 individuals passed the quality control criteria and were retained for the subsequent analyses.

The genomic data (sire genotypes) were explored for underlying population substructure using multi-dimensional scaling based on the genomic kinship matrix estimated from all SNPs in the analysis. The genomic kinship matrix was calculated as outlined by Amin et al. [26].

Subsequently, three alternative approaches were used to test for associations of genotypes with bTB susceptibility traits: GWA, RHM and chromosome association analyses. Each bTB trait was analysed separately. Prior to the association analyses, deregressed EBVs were weighted using the formula outlined by Garrick et al. [24]:

$$ {\omega}_i=\frac{1-{h}^2}{\left[ c+\left(1-{r_i}^2\right)/{r_i}^2\right]{h}^2} $$

where ω_*i*_ is the weighting factor of the deregressed EBV of the *i*th animal; h^2^ is the heritability of the trait (h^2^ = 0.09 [15]); r_*i*_^2^ is the reliability of the deregressed EBV of the *i*th sire and c is the genetic variance not accounted for by the SNPs. A value of 0.20 [27] was considered for c.

Furthermore, Pearson correlations between the three sets of sire EBVs were calculated.

### Genome-wide association analysis

GWA analysis was performed by regressing the deregressed EBV on each individual SNP using the following model:

1 $$ y=\mu + X b+ Z a+ e $$

where y is a vector of observations on the trait (de-regressed bull EBV); μ is the population mean; b is a vector of SNP fitted as a fixed effect; a is a vector of additive polygenic random effect including the genomic relationship matrix among individual animals; X and Z are incidence matrices for fixed effects and random effects, respectively; and e is the vector of residuals.

GWA analyses were conducted with the R-based statistical package GenABEL [28]. After Bonferroni correction, the genome-wide significant threshold (*P* = 0.05) was defined at *P* = 1.43 × 10^−6^ which corresponds to a –log_10_(P) = 5.84, whereas the suggestive threshold (i.e. one false positive per genome scan) was defined at *P* = 2.87 × 10^−5^ corresponding to a –log_10_ (P) = 4.54. The *P*-values obtained from the GWA analysis were adjusted for inflation using the genomic inflation factor, λ, which accounts for any systematic deviation of observed from expected *P*-values. The estimated polygenic heritability was calculated as h^2^ = (σ^2^_a_/σ^2^_p_) in which the phenotypic variance (σ^2^_p_) was obtained by summing the additive genetic (σ^2^_a_) and residual variance (σ^2^_e_) from model 1.

SNPs found to be significant in the previous step were further tested by fitting the respective genotypes individually as a fixed effect in a mixed model similar to model 1. These analyses were conducted with the ASReml software package [29]. The genotypic effect solutions were used to estimate the additive and dominance effects of the respective loci. The proportion of genetic variance of each trait explained by each SNP was estimated using the following equation:

$$ \mathrm{Proportion}\ \mathrm{of}\ \mathrm{genetic}\ \mathrm{variance}\ \mathrm{explained}\ \mathrm{b}\mathrm{y}\ \mathrm{S}\mathrm{N}\mathrm{P} = \left[2 pq{\left( a + d\left( q- p\right)\right)}^2\right]/{\sigma^2}_a $$

where a, d, p and q were respectively additive effects, dominance effects, allele frequencies at the SNP locus and σ^2^_a_ is the total genetic variance of the trait calculated with model 1 excluding the SNP effect.

Significant SNPs were also explored for linkage disequlibrium (LD) with other nearby SNPs. Pairwise LD, measured with r^2^ was calculated in the software PLINK [25] with LD and haplotype blocks visualised in Haploview software [30]. The haplotype blocks were identified using Wang’s method [31]. QTL regions surrounding significant SNPs were defined by the farthest neighbouring SNPs that had a minimum LD of 0.40 with the significant SNP in question. Subsequently, in order to identify candidate genes, the QTL regions were then matched onto the bovine reference genome that is publicly available through the *Bos_taurus_UMD_3.1.1* project of the National Centre for Biotechnology Information [32].

### Regional heritability mapping

The same data described above were analysed with the RHM approach, in which genomic regions of 100 SNPs were defined by sliding ‘windows’ shifting every 50 SNPs along each autosomal chromosome. A detailed description of RHM was given by [33].

The following model was applied for the RHM:

2 $$ y=\mu + X b+ Z a+ Z r+ e $$

where r is a vector of region (consisting of 100 SNPs) fitted as a random effect; with other terms in the model defined as in model (1).

RHM analyses were performed using the DISSECT software [34]. The significance of genomic regions was assessed with the likelihood ratio test (LRT) statistic, which was used to compare model (2) that fitted a genomic region as a random effect against the base model that excluded this effect. The LRT was derived as twice the difference between the log-likelihoods of the model including and excluding the regions in question. A total of 713 regions were tested across the genome, of which half were used in the Bonferroni correction to account for the shifting of regions every 50 SNPs. The LRT thresholds were 13.20 (*P* = 1.40 × 10^−4^) and 8.93 (*P* = 2.80 × 10^−3^) for the genome-wide and suggestive significance thresholds, respectively. The phenotypic variance was calculated as σ^2^_p_ = σ^2^_a_ + σ^2^_r_ + σ^2^_e_, while the regional (r) heritability was subsequently estimated as h^2^_r_ = σ^2^_r_/σ^2^_p_.

### Chromosome association analysis

In a separate set of analyses, the entire autosomal chromosome effect was fitted in model 2 instead of genomic region. After Bonferroni correction, the LRT significance thresholds for the genome-wide and suggestive levels were 8.55 (*P* = 1.72 × 10^−3^) and 4.47 (*P* = 3.45 × 10^−2^), respectively. The phenotypic variance was calculated as σ^2^_p_ = σ^2^_a_ + σ^2^_c_ + σ^2^_e_, where σ^2^_c_ was the variance due to the chromosomal genetic effect. The chromosomal (c) heritability was subsequently estimated as h^2^_c_ = σ^2^_c_/σ^2^_p_.

## Results

The multi-dimensional scaling analysis indicated that the sample population was homogenous, manifested by a single cluster of individuals (Additional file 1). The mean de-regressed EBVs for susceptibility to bTB among the traits ranged from 0.38 to 0.47 with mean reliabilities of deregressed EBVs ranging between 0.69 and 0.74 (Additional file 2). Correlation between sire de-regressed EBVs was high between phenotypes 2 and 3 (0.99), and lower between phenotypes 1 and 2 (0.54) and between phenotypes 1 and 3 (0.57).

### GWA analysis

**Fig. 1:**
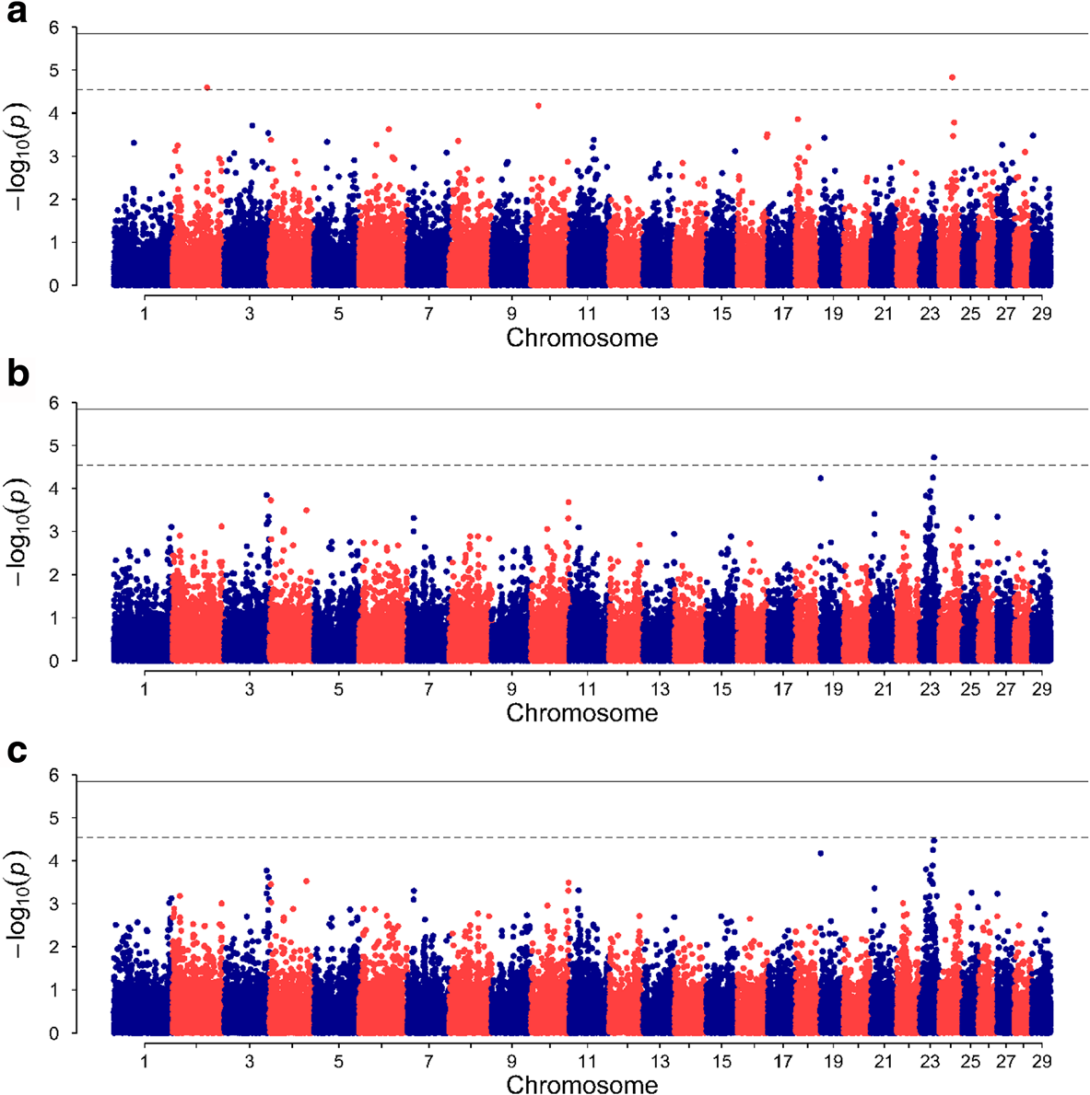
Manhattan plots displaying results of genome-wide association analyses of three bovine tuberculosis susceptibility traits. **a** phenotype 1, positive reactors to the skin test with positive post-mortem results; **b** phenotype 2, positive reactors to the skin test regardless of post-mortem results; **c** phenotype 3, as phenotype 2 plus non-reactors and inconclusive reactors with positive post-mortem examination results. Dashed and solid lines represent suggestive and genome-wide thresholds, respectively

Association between individual SNPs and bTB susceptibility traits are illustrated in the Manhattan plots in Fig. 1, with corresponding quantile-quantile plots in Additional file 3. Estimated polygenic heritability for the three bTB traits was moderate and ranged from 0.26 ± 0.07 to 0.37 ± 0.07, with heritabilities for phenotypes 2 and 3 being similar but both a little higher than for phenotype 1 (Additional file 2).

We identified three suggestive SNPs associated with the studied traits (Table 1). Two of these SNPs, ARS-BFGL-NGS-40833 (*P* = 2.56 × 10^−5^) and Hapmap38114-BTA-57971 (*P* = 1.48 × 10^−5^) were associated with phenotype 1 on BTA 2 and 24, respectively. The other SNP, BTA-56563-no-rs (*P* = 1.99 × 10^−5^) on BTA 23 was associated with phenotype 2. The SNP identified to affect phenotype 2 also reached but did not exceed the suggestive significance threshold for phenotype 3 (Fig. 1).

**Table 1:** SNPs identified in the genome-wide association analysis to be significantly associated with bovine tuberculosis traits. Phenotype 1, positive reactors to the skin test with positive post-mortem results; phenotype 2, positive reactors to the skin test regardless of post-mortem results

Phenotype	SNP name	BTA	Position	*P*-value
1	ARS-BFGL-NGS-40833	2	93065483	2.56 × 10^−5^
	Hapmap38114-BTA-57971	24	35403612	1.48 × 10^−5^
2	BTA-56563-no-rs	23	38412668	1.99 × 10^−5^

Additive and dominance effects of these SNPs and the proportion of the genetic variance explained by them are shown in Additional file 4. SNPs on BTA 2 and 23 had significant (P < 0.01) additive effects on phenotypes 1 and 2, respectively. However, there was no significant additive effects found for the SNP on BTA 24. The additive (allele substitution B to A) effect for the SNP on BTA 2 was 0.57 and the SNP accounted for 14% of the total genetic variance of susceptibility to bTB as defined by phenotype 1. The SNP on BTA 23 had an additive (allele substitution B to A) effect of 0.81 and explained 3% of the genetic variance of susceptibility to bTB as defined by phenotype 2. In both cases, the minor allele A was associated with increased resistance to bTB infection. No significant dominance effects (P > 0.05) were found for any SNP locus.

Putative QTL regions were defined based on the LD of our two significantly additive SNPs with neighbouring SNPs. The LD structure for these regions is presented in Additional files 5 and 6 for SNPs on BTA 2 and 23, respectively. The SNP on BTA 2 was located within a QTL region spanning 1.29 Mb. One relevant gene in the bovine reference genome found within this region, *PARD3B*, was about 157 Kb upstream of the SNP. The SNP identified on BTA 23 was located within a QTL region covering 1.2 Mb. The most relevant gene found in the region was *RNF144B,* located upstream of BTA-56563-no-rs.

Overall, the GWA analysis results showed that, although some SNPs are significantly associated with the traits of study, a considerable proportion of the genetic variance still remains unaccounted for. This is expected for traits with largely complex polygenic architectures.

### RHM analysis

**Fig. 2:**
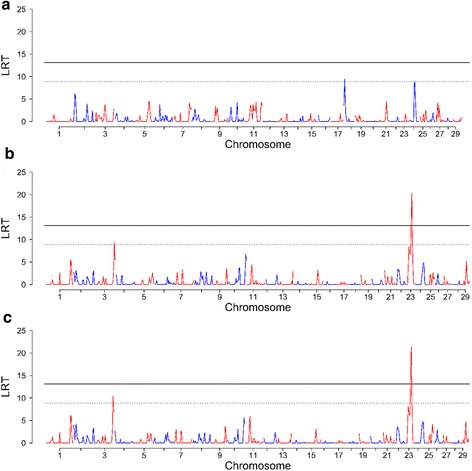
Manhattan plots displaying results of regional heritability mapping analyses of three bovine tuberculosis susceptibility traits. **a** phenotype 1, positive reactors to the skin test with positive post-mortem results; **b** phenotype 2, reactors to the skin test regardless of post-mortem results; **c** phenotype 3, as phenotype 2 plus non-reactors and inconclusive reactors with positive post-mortem examination results. Dashed and solid lines represent suggestive and genome-wide thresholds, respectively

The RHM analysis revealed two regions that crossed the genome-wide significance threshold for phenotypes 2 and 3 on BTA23 (Table 2; Fig. 2). Additional regions reached the suggestive significance threshold on BTA 3, 18 and 23 across the three traits (Table 2; Fig. 2).

**Table 2:** Genomic regions identified with regional heritability mapping (100-SNP windows) affecting three bovine tuberculosis traits. Phenotype 1, positive reactors to the skin test with positive post-mortem results; phenotype 2, positive reactors to the skin test regardless of post-mortem results; phenotype 3, as phenotype 2 plus non-reactors and inconclusive reactors with positive post-mortem examination results

Phenotype	BTA	Genomic regions (SNP name and position (bp))		LRT	h^2^_r_(SE)
		Start	End		
1	18	Hapmap57004-rs29011610	ARS-BFGL-NGS-1116	9.41^b^	0.06(0.03)
		4463083	9539002		
2	23	ARS-BFGL-NGS-78313 30222836	BTA-56563-no-rs 38412668	15.12^a^	0.05(0.03)
	23	ARS-BFGL-NGS-107881 33961556	BTB-00870908 41672507	20.41^a^	0.07(0.03)
	23	Hapmap31420-BTA-137383 38521106	ARS-BFGL-NGS-41732 44897933	9.27^b^	0.05(0.03)
	3	ARS-BFGL-84593 114388249	ARS-BFGL-NGS-26427 119113936	9.48^b^	0.07(0.03)
3	23	ARS-BFGL-NGS-78313 30222836	BTA-56563-no-rs 38412668	15.98^a^	0.05(0.03)
	23	ARS-BFGL-NGS-107881 33961556	BTB-00870908 41672507	21.37^a^	0.08(0.03)
	23	Hapmap31420-BTA-137383 38521106	ARS-BFGL-NGS-41732 44897933	9.76^b^	0.05(0.03)
	3	ARS-BFGL-84593 114388249	ARS-BFGL-NGS-26427 119113936	10.37^b^	0.08(0.04)

*h* ^*2*^_*r*_ regional heritability, *SE* standard error

^a^Genome-wide significance level; ^b^Suggestive significance level

Three overlapping regions were identified on BTA 23 affecting both phenotype 2 and 3: region 1 (30.2 - 38.4 Mb), region 2 (33.9 - 41.6 Mb) and region 3 (38.5 - 44.8 Mb). The SNP identified on BTA 23 with the GWA analysis was located within regions 1 and 2. The regional heritability estimates ranged from 0.05 to 0.08 (Table 2).

Two new significant regions on BTA 3 and 18, associated with phenotypes 2 and 3, and phenotype 1, respectively, were revealed. The GWA analysis had not identified any significant SNPs in these regions. Corresponding regional heritability estimates ranged between 0.06 and 0.08 (Table 2).

Another region on BTA 24 associated with phenotype 1, within which the SNP identified with the GWA analysis had been located, was just below the suggestive threshold of RHM (Fig. 2).

### Chromosome association analysis

The chromosomal association study (Additional file 7) revealed that BTA 23 had the greatest impact on phenotypes 2 and 3, and the highest LRT of 15.88 and 15.26, respectively. This is consistent with the GWA and RHM results. Corresponding chromosomal heritability estimates were 0.07 ± 0.03 and 0.08 ± 0.04 for the two traits, suggesting the regions identified with RHM in the present study were entirely responsible for this chromosome’s effect.

Regarding phenotype 1, the highest significant LRT was observed on a different chromosome (BTA 11), where neither GWA nor RHM analyses had revealed any significant associations. The corresponding chromosomal heritability was 0.08 ± 0.04 and was probably due to an aggregation of moderate effects of different genomic regions along this chromosome. Similarly, neither BTA 18 nor BTA 24, where RHM had revealed genomic regions with suggestive effects, reached a significance level in the chromosomal association analysis of phenotype 1 (Additional file 7).

## Discussion

Our results offer insights into the genomic architecture of susceptibility to bTB in British Holstein-Friesian dairy cattle. This is the first genomic study of this population that explores three different case phenotypes based on the bTB testing regime undertaken in Great Britain. In all cases, we used de-regressed sire EBVs as phenotypes. The latter are considered robust phenotypes for genomic analyses [23, 24, 35], representing the aggregate adjusted records for disease incidence of multiple progeny per sire.

The findings of the present study collectively suggest that considerable heritable variation at the genomic level influences differences in the inherent bTB susceptibility among animals. We found that heritability for bTB susceptibility was moderately high in this population and therefore selection for resistance is a feasible strategy to reduce the incidence of bTB nationwide. Other studies [12–16] corroborate these findings. Tsairidou et al. [13] and Bermingham et al. [19], respectively reported polygenic heritabilities of 0.23 and 0.21 for susceptibility to bTB, which were similar to the estimate for phenotype 1 in our study, based on positive skin test reactors with positive post mortem examination results. However, these heritability estimates were lower than those obtained for phenotypes 2 and 3 in the present study; these two trait definitions account for skin test imperfections and therefore, are likely to represent a different phenotype compared to conventionally confirmed cases. This finding is further supported by the relatively lower correlations between sire EBVs for phenotype 1 and those of the other two traits, which are in agreement with results from Banos et al. [15].

GWA analysis conducted in the present study identified two QTL regions that may influence animal susceptibility to bTB. The global Holstein-Friesian cattle population has high levels of genetic relatedness among animals (population structure) manifested by a small effective population size, which may result in false associations [36]. However, in the present study, inclusion of the genomic relationship matrix in the model accounted for the population structure. Relatively few individual SNPs with a significant effect on the bTB traits were identified through GWA analysis. This could be explained by the complex genetic architecture underlying susceptibility to bTB and the polygenic nature of the disease as suggested by Bermingham et al. [19]. It could also be partly attributed to the conservativeness of the Bonferroni correction method used to adjust for multiple testing, which often inflates type II errors [37].

The present study identified two additive SNPs in moderate LD with neighbouring SNPs on BTA 2 and 23 that were significantly associated with different traits of susceptibility to bTB. In both cases, the allele with the minor frequency had the favourable additive effect, conferring increased resistance to bTB in the studied population. A similar result reported by Bermingham et al. [19] indicated that the major frequency alleles of SNPs on BTA 2 (different region compared to our study) and 13 were associated with a greater risk of bTB infection. Richardson et al. [18], however, found that the major frequency alleles of SNPs located on BTA 1 and 23 (different region compared to our study) were associated with bTB resistance. In all cases, different SNPs and cattle populations are involved. The SNPs identified in the present study provide possible markers for selecting against susceptible individuals with the potential to improve inherent resistance to the disease in the British Holstein population.

The length of the putative QTL regions defined in the present study (1.20-1.29 Mb) was similar to those reported by Kim and Kirkpatrick [38] where the median physical distance between pairs of markers at a mean LD of 0.48 was about 1.13 Mb in Holstein cattle. We identified candidate genes within these regions with possible underlying effects on disease susceptibility. The significant SNP on BTA 2 was located close to gene *PARD3B*, which has been implicated in protection against disease progression in patients affected by the human immune deficiency virus and acquired immune deficiency syndrome (HIV/AIDS) [39]. Similarly to bTB in cattle, HIV/AIDS is a chronic, progressive illness of humans. The most relevant gene close to the SNP on BTA 23 was *RNF144B*. This protein coding gene has been found to play a role in the regulation of NF-κB in human macrophages. NF-κB regulates the expression of various genes involved in diverse cellular processes including inflammation and immunity [40] and has been associated with endometriosis in humans [41]. Other functions of the *RNF144B* gene include roles in regulation of apoptosis and cell proliferation, making the gene a possible candidate for therapeutic treatment of endometrial cancer [42]. Further studies based on expression profiles and pathway analyses may shed more light into the function of the above genes in relation to cattle susceptibility to bTB.

The present study did not confirm QTL identified in previous association studies on bTB susceptibility [17–21], which further supports the notion of a polygenic trait controlled by multiple genes. The closest GWA results on BTA 23 were reported by Richardson et al. [18] who identified a QTL about 28 Mb downstream on the same chromosome for Irish dairy cattle. Richardson et al. [18] also used de-regressed EBVs based on a phenotype similar to phenotype 2 in our study.

The RHM analysis overcame some of the limitations of GWA due to the former’s capacity to consolidate a proportion of genomic variation based on multiple neighbouring marker effects [33]. In the present study, RHM identified significant new genomic regions on BTA 18 for phenotype 1 and BTA 3 for phenotypes 2 and 3, where GWA had not identified individual SNPs with a significant effect on the respective traits. This suggests that RHM may identify regions harbouring individual SNPs with moderate or even non-significant effects, which, however, may collectively have a significant impact on bTB susceptibility. Importantly, RHM also identified significant genomic regions including the individual SNPs with a significant effect in the GWA analysis, thereby corroborating the suggestion of a QTL presence. The three genomic regions identified on BTA 23 support the possibility of a large region with overlapping genetic variants. RHM has previously been used in association studies of susceptibility to bTB in a different cattle population [19, 43]. Although no common regions with those of our study were reported, Wilkinson et al. [43] identified a region further downstream (at 6.6 - 7.1 Mb) of our region on BTA 23 affecting positive reactors to the skin test with negative post-mortem results (unconfirmed cases).

Furthermore, the present study has highlighted a major overall chromosomal influence of BTA 23 on susceptibility to bTB, when the definition of the latter is not restricted to post-mortem confirmed cases but includes all positive skin test reactors and all animals with a positive post-mortem result. Actually, chromosome 23 was the only chromosome that featured in the significant results of all our analyses (GWA, RHM, chromosomal association). Notably, BTA 23 harbours the major histocompatibility complex (MHC), which plays a central role in immune response to infection [44, 45]. Our region was located about 10 Mb upstream of the MHC region based on GWA and 2 Mb based on RHM results. In addition, Zare et al. [46] found genomic regions on BTA 23 (at 35.3 and 44.4 Mb) associated with paratuberculosis in Jersey cattle, a disease with certain similarities to bTB. These regions corresponded to our RHM identified regions on BTA 23.

Previous genomic studies on cattle susceptibility to bTB have not resulted in consistent outcomes to support a common genomic mechanism underlying the trait. Some of our results might have added to the wealth of diverse findings. As discussed, reasons for such discrepancies include the complexity of the phenotype, the largely polygenic inheritance mode of the trait, genetic differences between populations and differences in methodologies used across studies. Additional reasons may be different allele frequencies of either the marker or causative mutation even when the same QTL is segregating in various populations, and possible mutation linkage phases that may not be the same between populations [20, 47]. Moreover, bTB is an infectious disease whose profile and transmission dynamics may differ across populations and geographic regions, thereby further complicating the genomic study of the underlying control mechanism. All these reasons together suggest that scientific results are likely to be relevant primarily to the studied population and trait definitions on which they were based.

## Conclusions

Our results suggest that bTB susceptibility in the British Holstein cattle population is a moderately heritable polygenic trait, potentially amenable to improvement with selective breeding. Our findings may inform genomic predictions (genomic EBV calculations) within a genomic selection programme, where differential emphasis can be placed on specific genomic regions identified to have significant effects on the trait. At the same time, it would be useful to quantify the impact of such a selection process on the disease dynamics as well as other traits of the breeding goal. Our results may also provide target areas for possible future gene editing applications within a genetic improvement programme.

## Additional files

Additional file 1: Multi-dimensional scaling (Principal Component) analysis of an identity by state matrix of 804 bulls. A single cluster was formed which reflect homogeneity of the population. (DOCX 407 kb)

Additional file 2: Genetic parameters of three bovine tuberculosis traits. (DOCX 14 kb)

Additional file 3: Quantile-quantile plots of observed against expected *P*-values from genome-wide association analyses: (a) phenotype 1, positive reactors to the skin test with positive post-mortem results; (b) phenotype 2, positive reactors to the skin test regardless of post-mortem results; (c) phenotype 3, as phenotype 2 plus non-reactors and inconclusive reactors with positive post-mortem examination results. (DOCX 1200 kb)

Additional file 4: Additive and dominance effects for significant SNPs identified by genome-wide association analysis. (DOCX 13 kb)

Additional file 5: Linkage disequilibrium (r^2^) map of a QTL region on BTA 2 affecting bTB (phenotype 1). The region ranges from SNP ARS-BFGL-NGS-40833 (bp = 93065483) to SNP ARS-BFGL-NGS-109114 (bp = 94352603); white for r^2^ = 0, shades of grey for 0 < r^2^ < 1 and black for r^2^ = 1. (DOCX 54 kb)

Additional file 6: Linkage disequilibrium (r^2^) map of a QTL region on BTA 23 affecting bTB (phenotype 2). The region ranges from SNP ARS-BFGL-NGS-88425 (bp = 38206814) to SNP BTA-01409-rs29012374 (bp = 39411428); white for r^2^ = 0, shades of grey for 0 < r^2^ < 1 and black for r^2^ = 1. (DOCX 57 kb)

Additional file 7: Manhattan plots displaying results of chromosomal association analyses of three bovine tuberculosis susceptibility traits: (a) phenotype 1, positive reactors to the skin test with positive post-mortem results; (b) phenotype 2, positive reactors to the skin test regardless of post-mortem results; (c) phenotype 3, as phenotype 2 plus non-reactors and inconclusive reactors with positive post-mortem examination results. Dashed and solid lines represent suggestive and genome-wide thresholds, respectively. (DOCX 2254 kb)

## Abbreviations

BTA: *Bos taurus* autosome
bTB: Bovine tuberculosis
EBV: Estimated breeding value
GWA: Genome-wide association
LD: Linkage disequilibrium
LRT: Likelihood ratio test
QTL: Quantitative trait loci
RHM: Regional heritability mapping
SNP: Single neucleotide polymorphism
