# Supplementary figures and images for: Genomic regions underlying susceptibility to bovine tuberculosis in Holstein-Friesian cattle
Source: BMC Genet. 2017 Mar 23;18:27. doi: 10.1186/s12863-017-0493-7 (PMC5364629; doi:10.1186/s12863-017-0493-7)

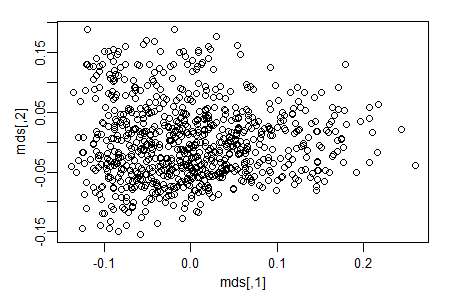

Supplement: Supplementary file 1 — Multi-dimensional scaling (Principal Component) analysis of an identity by state matrix of 804 bulls. A single cluster was formed which reflect homogeneity of the population. (DOCX 407 kb) [file 12863_2017_493_MOESM1_ESM.docx]

**a**


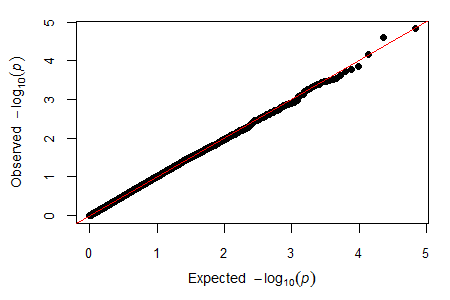


**b**


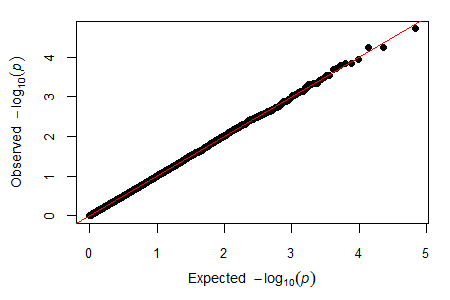


**c**


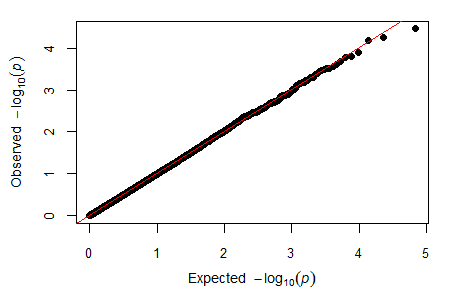

Supplement: Supplementary file 3 — Quantile-quantile plots of observed against expected P-values from genome-wide association analyses: (a) phenotype 1, positive reactors to the skin test with positive post-mortem results; (b) phenotype 2, positive reactors to the skin test regardless of post-mortem results; (c) phenotype 3, as phenotype 2 plus non-reactors and inconclusive reactors with positive post-mortem examination results. (DOCX 1200 kb) [file 12863_2017_493_MOESM3_ESM.docx]

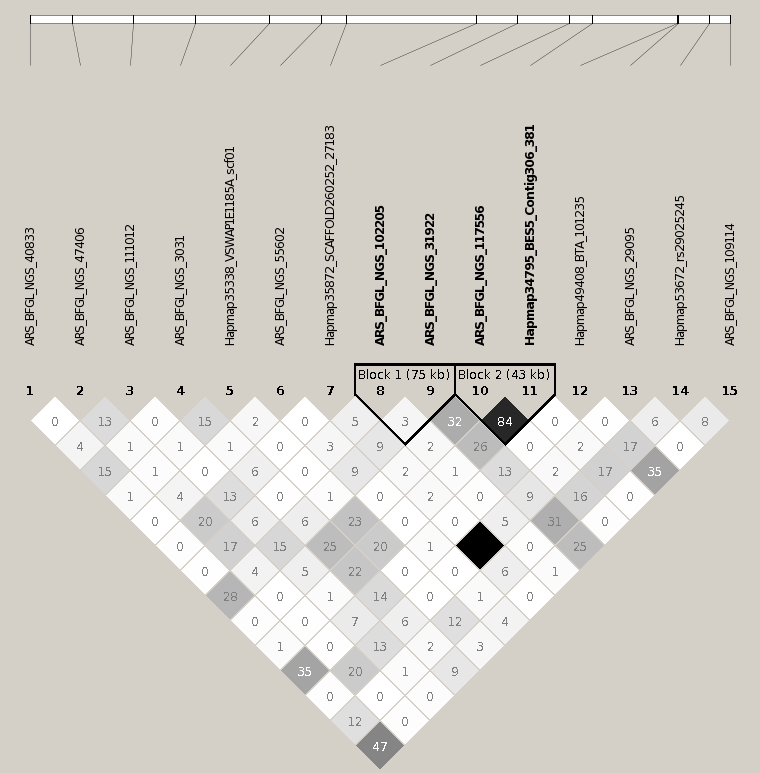

Supplement: Supplementary file 5 — Linkage disequilibrium (r2) map of a QTL region on BTA 2 affecting bTB (phenotype 1). The region ranges from SNP ARS-BFGL-NGS-40833 (bp = 93065483) to SNP ARS-BFGL-NGS-109114 (bp = 94352603); white for r2 = 0, shades of grey for 0 < r2 < 1 and black for r2 = 1. (DOCX 54 kb) [file 12863_2017_493_MOESM5_ESM.docx]

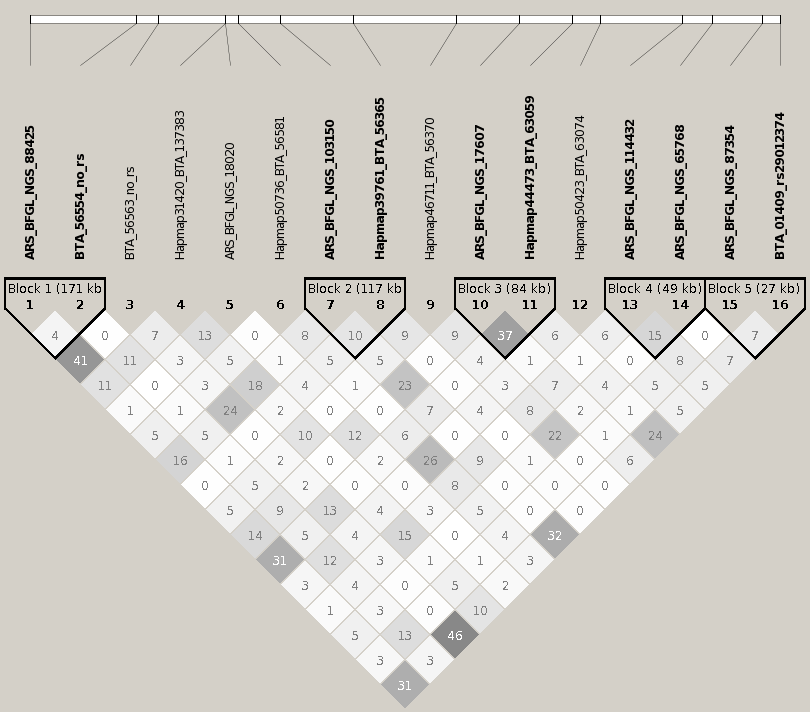

Supplement: Supplementary file 6 — Linkage disequilibrium (r2) map of a QTL region on BTA 23 affecting bTB (phenotype 2). The region ranges from SNP ARS-BFGL-NGS-88425 (bp = 38206814) to SNP BTA-01409-rs29012374 (bp = 39411428); white for r2 = 0, shades of grey for 0 < r2 < 1 and black for r2 = 1. (DOCX 57 kb) [file 12863_2017_493_MOESM6_ESM.docx]

**a**
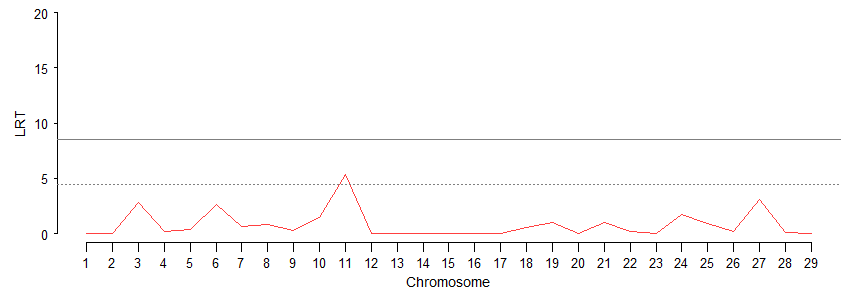


**b**
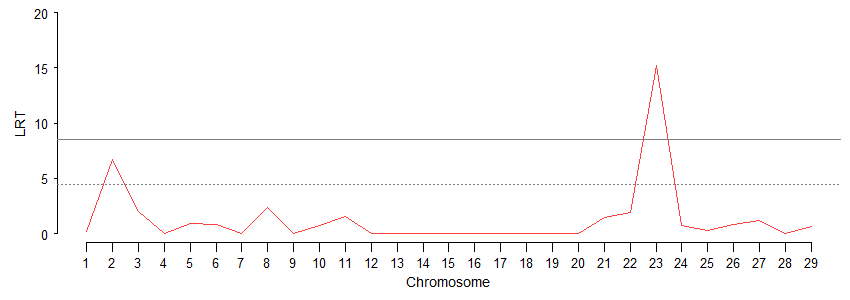


**c**
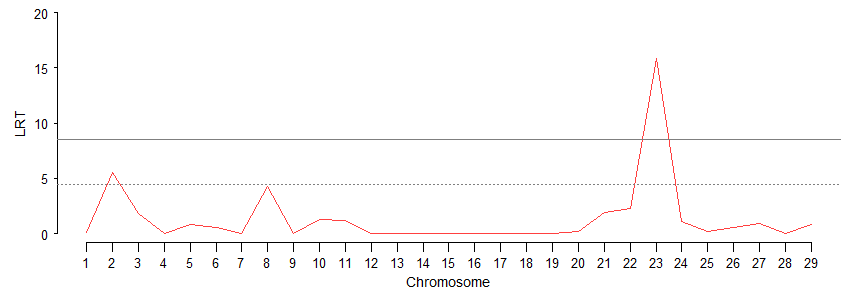

Supplement: Supplementary file 7 — Manhattan plots displaying results of chromosomal association analyses of three bovine tuberculosis susceptibility traits: (a) phenotype 1, positive reactors to the skin test with positive post-mortem results; (b) phenotype 2, positive reactors to the skin test regardless of post-mortem results; (c) phenotype 3, as phenotype 2 plus non-reactors and inconclusive reactors with positive post-mortem examination results. Dashed and solid lines represent suggestive and genome-wide thresholds, respectively. (DOCX 2254 kb) [file 12863_2017_493_MOESM7_ESM.docx]
