# Supplementary material for: Genomic regions underlying susceptibility to bovine tuberculosis in Holstein-Friesian cattle
Source: BMC Genet. 2017 Mar 23;18:27. doi: 10.1186/s12863-017-0493-7 (PMC5364629; doi:10.1186/s12863-017-0493-7)
Supplement: Supplementary file 2 — Genetic parameters of three bovine tuberculosis traits. (DOCX 14 kb) [file 12863_2017_493_MOESM2_ESM.docx]

**Additional file 2** Genetic parameters of three bovine tuberculosis traits

| Phenotype^1^ | Mean de-regressed EBV | Mean reliability of sire EBV | Polygenic heritability (SE) |
| --- | --- | --- | --- |
| 1 | 0.38 | 0.69 | 0.26 (0.07) |
| 2 | 0.44 | 0.74 | 0.37 (0.07) |
| 3 | 0.47 | 0.74 | 0.34 (0.07) |

^1^Phenotype 1, positive reactors to the skin test with positive post-mortem results;

phenotype 2, positive reactors to the skin test regardless of post-mortem results;

phenotype 3, as phenotype 2 plus non-reactors and inconclusive reactors with positive

post-mortem examination results

EBV = estimated breeding value; SE = standard error
