# Supplementary material for: Genomic regions underlying susceptibility to bovine tuberculosis in Holstein-Friesian cattle
Source: BMC Genet. 2017 Mar 23;18:27. doi: 10.1186/s12863-017-0493-7 (PMC5364629; doi:10.1186/s12863-017-0493-7)
Supplement: Supplementary file 4 — Additive and dominance effects for significant SNPs identified by genome-wide association analysis. (DOCX 13 kb) [file 12863_2017_493_MOESM4_ESM.docx]

**Additional file 4** Additive and dominance effects for significant SNPs identified by genome-wide association analysis.

| Phenotype^1^ | SNP | Allele frequency  p q | | P-value (SNP) | a (SE) | P-value (a) | d (SE) | P-value (d) | VA_prop_ |
| --- | --- | --- | --- | --- | --- | --- | --- | --- | --- |
| 1 | SNP1 | 0.63 | 0.37 | 0.001 | 0.57 (0.14) | 7.74x10^-5^ | -0.05 (0.17) | 0.38 | 0.14 |
|  | SNP2 | 0.89 | 0.11 | 0.001 | 0.66 (0.36) | 7.90x10^-2^ | 0.23 (0.40) | 0.34 | 0.04 |
| 2 | SNP3 | 0.84 | 0.16 | 0.001 | 0.82(0.32) | 1.57x10^-2^ | 0.31 (0.36) | 0.27 | 0.03 |

^1^Phenotype: phenotype 1: positive reactors to the skin test with positive post-mortem results; phenotype 2, all reactors to the skin

test regardless of post-mortem results.

Allele frequency: p and q; a = additive genetic effect; d = dominance effect; SE = standard error;

VA_prop_ = proportion of genetic variance due to SNP, where VA is the total additive genetic variance estimated from a

model ignoring SNP effects;

SNP1 = ARS-BFGL-NGS-40833; SNP2= Hapmap38114-BTA-57971; SNP3 = BTA-56563-no-rs.
